# A Non-Invasive Hair Test to Determine Vitamin D_3_ Levels

**DOI:** 10.3390/molecules26113269

**Published:** 2021-05-28

**Authors:** Iltaf Shah, Mohammad Mansour, Sheikh Jobe, Emadaldeen Salih, Declan Naughton, Syed Salman Ashraf

**Affiliations:** 1 Department of Chemistry, College of Science, UAE University, Al Ain P.O. Box 15551, United Arab Emirates; 201350291@uaeu.ac.ae (M.M.); 201350534@uaeu.ac.ae (S.J.); 201250376@uaeu.ac.ae (E.S.); 2 School of Life Sciences, Pharmacy and Chemistry, Kingston University London, Surrey KT1 2EE, UK; D.Naughton@kingston.ac.uk; 3 Department of Chemistry, College of Arts and Sciences, Khalifa University, Abu Dhabi P.O. Box 127788, United Arab Emirates

**Keywords:** vitamin D assay, hair analyses, liquid chromatography-mass spectrometry

## Abstract

Vitamin D deficiency is being recognized as a global issue and has been implicated in many health issues. Hence, there is an increased interest in developing sensitive, reproducible, and non-invasive assays to measure Vitamin D levels. This study aimed to apply a sensitive liquid chromatography-mass spectrometric assay to hair samples to develop and validate a clinical assay to provide a quarterly average level of vitamin D in one test. Hair samples were collected from 70 male university students/young adults and pulverized/sonicated in methanol/water for 2 h to extract Vitamin D metabolites. A sensitive liquid chromatographic-mass spectrometric assay was employed to quantitate vitamin D and metabolites. Of the eight Vitamin D and metabolites screened, only the primary, clinically significant form of vitamin D (25OHD_3_) was detected and quantified in hair samples in the range of 17–1541 pg/mg. One-third of the hair samples (21 out of 70) had Vitamin D levels below the LLOD of the assay (10 pg/mg). The mean and standard deviation values for hair (25OHD_3_) were 276.7 ± 329.9, respectively. This pilot study reveals the potential of the vitamin D hair test in clinical assays as a complementary test to a vitamin D blood test, which would provide a quarterly average.

## 1. Background

It is estimated that nearly 1 billion people are vitamin D deficient worldwide, either due to reduced sun exposure or insufficient dietary intake. Vitamin D deficiency is implicated in many diseases, but these associations lack clarity [1,2]. Studies on the role of vitamin D in health and disease have been hampered by the difficulty of the assays required, which, in turn, arises from the complex number of the vitamin D metabolites and issues in separating them for detection [3,4,5].

The endogenous and most studied form, vitamin D_3_, is synthesized in the skin via exposure of precursor 7-dehydrocholesterol to UVB radiation from sunlight [6,7]. Vitamin D_2_ is not naturally formed in humans; instead, it can be consumed via diet and supplementation. Vitamin D (D_3_ and D_2_) undergoes hydroxylation processes in the liver to form 25OHD_3_ and 25OHD_2_, respectively [8]. Further hydroxylation in the kidney generates the established active form 1α,25-dihydroxyvitamin D. The half-life of 25OHD forms is around 2 to 3 weeks as compared to the other metabolites, which are around 4 h. In recent years, a further complexity arose as epimeric forms of 25OHD_2_ and 25OHD_3_ have been identified and quantified in health and in various conditions [3,6,9,10]. To date, blood tests for the long-lived circulating form (25OHD) have been used as a gold standard to evaluate vitamin D deficiency.

The lack of association of vitamin D levels with the development and progression of a number of diseases has been the subject of considerable investigation. Recent advances in the capacity of vitamin D assays have focused on more accurate measurements of blood levels of the circulating forms (25OHD), with some extending the measurements to multiple forms of vitamin D [4,11,12]. Notable improvements include the separation and quantification of the epimer forms along with the ability to measure circulating and active forms simultaneously. These approaches will, for the first time, allow researchers to study the roles of a variety of forms of vitamin D and by accurate assays to relate the circulating forms to disease initiation, progression, and alleviation. In due course, it is expected that these advanced liquid chromatography-tandem mass spectrometric (LC-MS/MS) methods will be routinely available for clinical assessments of a range of biomarkers, including vitamin D forms.

With methods available to simultaneously quantify 10 forms of vitamin D in blood [13], further advances are most likely to arise from innovative approaches for the analysis of these metabolites in other body matrices. Current blood-based assays present a number of limitations. Owing to expense [14] and custom, an individual blood test is common, yet it provides only a single, time-point snapshot of a complex biomarker. This adds difficulty to any study investigating the putative association of vitamin D levels and/or supplementation with disease initiation or progression. In such trials, a wide range of confounding factors exist, ranging from variations in levels of vitamin D depending on diet, sun exposure, skin type, and metabolic differences [15].

Further complications arise with the use of the circulating forms of vitamin D (25OHD_2_ and 25OHD_3_) beyond the fact that they are precursors and not the active form. Several studies have failed to find a clear correlation between the levels of circulating and active forms [4,13,16,17,18]. It is known that the circulating forms of vitamin D (25OHD) are present at a comparatively higher concentration (nmol/L) as compared to the active forms of vitamin D (1α25(OH)_2_D) present at (pmol/L) and, furthermore, it is also known that metabolic and regulatory factors could also intercede to alter the levels of circulatory forms, which, in turn, will further compromise and reduce the concentrations of the active downstream forms of vitamin D [3]. Recently, using in vitro, cell-based studies, the epimer was shown to have a range of commensurate activities (coactivator peptide recruitment and anti-proliferative cellular effects) but also higher stability compared to the established active form [19]. Thus, the epimer forms have interfered with assays to date and, now that methods have been developed to discard their effect on accuracy, they appear to have an activity that cannot be disregarded for trials of supplementation of association of vitamin D and disease onset and progression.

A distillation of these issues points to a very complex field with an increasing number of questions rather than answers. In consequence, much debate surrounds the establishment of levels of vitamin D required for health maintenance. It is generally accepted that large parts of the Western world population are deficient in vitamin D [2,20], especially in dark winter months when little vitamin D_3_ can be made. However, agreement on acceptable levels of the circulating form 25OHD is far from in reach. For example, in the UK, the Scientific Advisory Committee on Nutrition and the Food Standards Agency define sub-optimal vitamin D as the circulating form (25OHD) being below 25 nmol/L [21]. In contrast, the US Institute of Medicine recommends vitamin D intake to give blood status to be <50 nmol/L [22]. Others have proposed that levels should be considerably higher, with optimal vitamin D status as being between 100–250 nmol/L and deficiency being defined as <120 nmol/L [23,24].

Given the range of issues surrounding any planned trial involving the association of vitamin D with the disease, it may be timely to look further afield in terms of biomarker status. With the continuous development of more sensitive instrumentation, the analysis of alternative biological samples is becoming a reality. One approach to overcome various barriers would be the development of a hair-based assay. Given that human cranial hair grows at a rate of some 1 cm per month, hair samples frequently offer an assay window of three months or more. The technology has been developed over recent years and is now admissible in a court of law for a range of forensic applications such as drug abuse. With the standardization of hair-based assays, the potential for biomedical tests for a range of biomarkers is considerable. As well as the advantage of hair samples providing measurements over several months, ease of sample collection, transport, storage, and reduced infection risks are also attractive. A recent study published preliminary results of an LC-MS-based hair test for a vitamin D (25(OH)D_3_) test conducted on a limited sample of three individuals [25].

Along with the methodological advances, equipment manufacturers have been developing much more sensitive mass spectrometers to extend the range of analytes that may be detected at physiologically relevant levels in a variety of matrices. Further advances in instrument software allow rapid analysis of a large number of analytes in a single scan lasting only minutes. This approach, termed Dynamic Multiple Reaction Monitoring, has been used to simultaneously measure several hundred drugs and their metabolites in minutes by focusing on selected peaks in an interval. Using this approach, 10 forms of vitamin D were quantified in blood in a 6-minute run time [4]. These combined advances suggest that a new era of biomedical analyses for small molecules is approaching.

The aims of this pilot study were (1) to develop and validate a hair-based assay method for eight forms of vitamin D (including 25OHD_3_) and (2) to trial the procedure as part of a larger study on university students.

## 2. Methods

### 2.1. Study and Participants

For the current study, 70 healthy Emirati male students (age 18–35) from UAE University were recruited in December 2019, and signed consent forms were obtained from all the volunteers, as per the UAE University ethical approval protocol number (UAEU Ref# SNA/fa/19-15). Around 500 mg of hair sample were cut directly from the vertex posterior of the head regions and kept in a labeled, sealed, plastic bag in a cold and dark place before sample processing.

### 2.2. Standards and Reagents

All reagents were of high-purity LCMS grade. Vitamin D_3_ (6,19,19-d3) as (internal standard), vitamin D_3_, vitamin D_2_, 25OHD_2_, 25OHD_3_, 1α25(OH)_2_D_2_, 1α25(OH)_2_D_3_, 3-epi-25OHD_3_, 3-epi-25OHD_2_, dichloromethane, methanol, deionized water, formic acid, ammonium formate, acetonitrile, and hexane were purchased from Emirates Scientific LLC Dubai, UAE.

### 2.3. Calibrant and Stock Solutions

Stock solutions of vitamin D analytes and internal standard Vitamin D_3_ (6,19,19-d3) were prepared by diluting and dissolving them in methanol solvent and acquiring a 1-mg/mL concentration. The standard mixture solutions were prepared by diluting the individual stock solutions Individual stock solutions were diluted to prepare a mixture of individual analytes in methanol arriving at a concentration of 1 μg/mL. Moreover, working solutions of the individual standards were also prepared in methanol at various concentrations. These solutions were then spiked at different concentrations in hair powder. Calibrants and quality control samples were prepared in blank human hair samples having no vitamin D forms detected. All solutions were stored in amber glass tubes at −20 °C until further analysis. Blank hair was selected from one of the 70 human hair samples (collected in large quantities). The selection of blank hair was carried out by collecting six different lots of presumed vitamin D-free human hair (collected in large quantities, i.e., full head shave). All hair samples were ground, and representative samples were collected from all six. The chosen representative sample was extracted and run on the LC-MS/MS method in triplicate as described in the Method section. It was found that there were no interferences from the matrix, and no vitamin D metabolites’ peaks were found in a few of the selected samples. One sample was chosen out of the six, which was the cleanest of the blank samples, with no interfering peaks that were expected at the retention time of any of the vitamin D metabolites’ peaks and hair matrix. The chosen blank human hair sample was selected for the preparation of the calibration curve and quality controls.

### 2.4. Sample Pre-Treatment

About 3-cm length of human hair corresponding to approximately 3 months of growth and about 500 mg of weight was washed with methanol/water mixture (this was to remove sweat sebum and exogenous hair product and contaminants) and then dried at room temperature under a gentle stream of N2. Then, the hair sample was pulverized into a fine powder using a mini ball mill (Fritsch and Gerhardt UK Ltd., Brackley, UK). Then, 200 mg of the ground hair was weighed in a weighing boat and transferred to a test tube. Washing was also collected and analyzed for any traces of vitamin D metabolites, but none was detected.

### 2.5. Sample Extraction

Standards and internal standard Vitamin D_3_ (6,19,19-d3) were added to the calibrants, quality controls, and to the hair samples under investigation, and they were sonicated in 2 mL methanol/water mixture (50:50; *v*/*v*) for 2 h, followed by centrifugation at 1250× *g* for 15 min. Then, the top layer was collected and filtered through a syringe filters’ PTFE membrane (0.45 μm) into new test tubes and then a gentle stream of N2 was used to dry-down the sample at 40 °C. We tried the extraction method with methanol alone followed by water alone and, when methanol was used together with water, got good extraction recovery (50:50; *v*/*v*). The sample was reconstituted in 50 µL of methanol: water (50:50, *v*/*v*), and 20 µL were injected into the LCMS/MS system.

### 2.6. LC-MS/MS System

The LC-MS/MS system is comprised of an 8060 tandem mass spectrometer, in combination with the Nexera ultra-high-pressure liquid chromatography (UHPLC) system (Shimadzu, Japan). The Nexera X2 UHPLC consisted of a modular system design with a pump, auto-sampler, column oven, and degasser. This system uses small particle columns and has a higher pressure tolerance. The auto-sampler has the capacity for high-speed injection, multi-solvent loops, and injection port rinsing. The mass spectrometer (MS) was operated using the positive electrospray ionization (ESI) mode. The LCMS-8060 is operated using Shimadzu’s Lab Solutions software that was used for instrument control, data handling, and analysis.

Ascentis Express F5 column, of dimensions 150mm × 2.1mm × 2.7 μm, was used for the chromatographic separation that was achieved. An HPLC guard column was connected to the above column for physical filtration. The column thermostat was kept at 50 °C. An injector wash program was used to rinse the needle with a methanol/water (75:25) mixture after each sample injection to minimize sample contamination. All mobile phases used were prepared from LC-MS grade solvents. Mobile phase A consisted of 5 mM of ammonium formate (0.315 g/L) in water (pH 6 adjusted with formic acid), and mobile phase B consisted of methanol with 5 mM of ammonium formate (0.315 g/L). The flow rate of the mobile phase was set to 0.5 mL/min with a binary gradient pump. The gradient elution program was used for optimum chromatography. Mobile phase B started at 80% from 0–4.0 min and linearly increased to 100% B; from 4.0–10 min it was kept at 100% B; from 10–14 min, before decreasing to initial conditions, it was 80% B. At 14.1–20.0 min., mobile phase A started at 20% and linearly decreased down to 0% A before increasing to 20% A again, at the timings shown above.

Positive electrospray ionization mode was used for the operation of mass spectrometer at a capillary temperature of about 350 °C and a spray voltage of about 4500 V. The drying gas flow was 10 L/min and nebulizing gas flow was set to 3 L/min.

The protonated molecules of analytes generated acted as precursor ions, which were broken down to produce ions during the collision-induced dissociation during MS/MS analysis. The precursor and product were measured together in multiple reaction monitoring (MRM) mode and were used for the analysis of all analytes. The most abundant MRM ion transitions for each analyte are given in Table 1 below.

### 2.7. Method Validation

The method was developed and validated for precision, accuracy, linearity, specificity, and recovery according to the US Food and Drug Administration (FDA) guidelines for method validation [26]. The linearity, intra- and inter-day precision, and accuracy of the vitamin D assays were calculated from analyzing the quality controls (QCs) at three different concentrations, namely, quality control low (QCL), quality control medium (QCM), and quality control high (QCH). QCL, QCM, and QCH were prepared at concentrations of 200 pg/mg, 400 pg/mg, and 1200 pg/mg, respectively. In each validation run, six QCs at each concentration level (QCL, QCM, and QCH) were analyzed along with a calibration curve. All quality control samples and calibration curves were prepared in human hair, having no 25OHD_3_ or any other vitamin D observed.

For the recovery experiment, six quality control samples at three different concentrations (QCL, QCM, and QCH) were spiked with vitamin D metabolites in methanol, and the neat quality controls at the three concentrations were dried down with a gentle stream of N2 and then reconstituted in methanol: water (50:50, *v*/*v*). The same experiment was repeated with blank hair samples spiked with quality controls at three similar concentrations. The hair samples were passed through the whole process of extraction, filtration, drying, and reconstitution as described in the Method section above and then compared using the peak area results (or area under the normal curve). The extracted and unextracted QCs’ values were employed to calculate the absolute percentage recovery using the following equation:

(1) $$\%\;\mathrm{Recovery}=\frac{\left(\mathrm{mean}\;\mathrm{extracted}\;\mathrm{QC}\;\mathrm{values}\right)}{\left(\mathrm{mean}\;\mathrm{unextracted}\;\mathrm{QC}\;\mathrm{values}\right)}\times 100$$

## 3. Results and Discussion

State-of-the-art LC-MS/MS instruments are very sensitive, offering multiple quantifications and verification points for each analyte. These characteristics confirm analyte identification and include the retention time on the LC column and the various fragmentation reactions that are characteristic of a particular molecule.

The method was validated for vitamin D_3_, vitamin D_2_, 25OHD_2_, 25OHD_3_, 1α25(OH)_2_D_2_, 1α25(OH)_2_D_3_, 3-epi-25OHD_3_, 3-epi-25OHD_2_, and inter-day and intra-day precision and accuracy values, along with recovery values that were calculated as summarized in Table 2 below. The lower limit of detection (LOD) was found to be 10 pg/mg for all analytes with a linear range from 15 pg/mg to 2000 pg/mg for all vitamin D metabolites. The linearity was determined by making sure that the linear regression (R^2^) values for the calibration curve were equal to or greater than 0.9996. The calibration curve was accepted with at least six calibrants on the curve, according to FDA guidelines.

**Figure 1:**
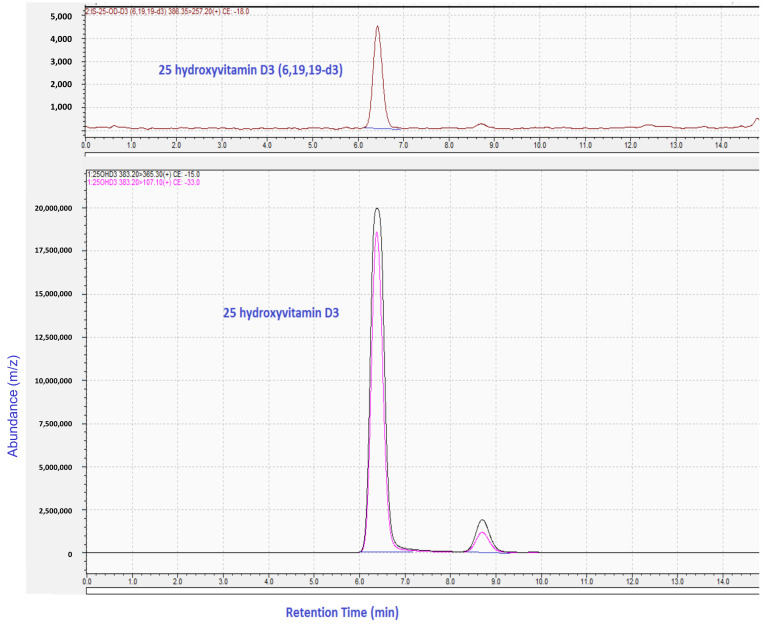
LC-MS/MS chromatogram showing retention time (top) and product ion mass spectrum of 25OHD_3_ extracted from human hair matrix under investigation. Figure 1: LC-MSMS MRM spectra for the internal standard, 25 hydroxyvitamin D_3_ (6,19,19-d3), [(386.35 → 257.20)], and 25 hydroxyvitamin D3 (383.20 → 365.3 and 383.30 → 107.10).

Also shown in Figure 1 are the precursor and product ion pairs and the optimized collision energies used for the MRM method.

Figure 1 shows the LC retention time and MRM used for the analysis of 25OHD_3_ in hair, in line with the validated procedure developed for hair samples. This key analyte normally accounts for some 80% of circulating forms and, as it is a precursor to the most active form (1α,25- dihydroxyvitamin D_3_). 25OHD_3_ provides a good foundation for the hair-based assay. The molar mass of 25OHD3 is 400.64 g/mol and during electrospray ionization it quickly loses water to give 383.2 [M+H]^+^ formation.

**Figure 2:**
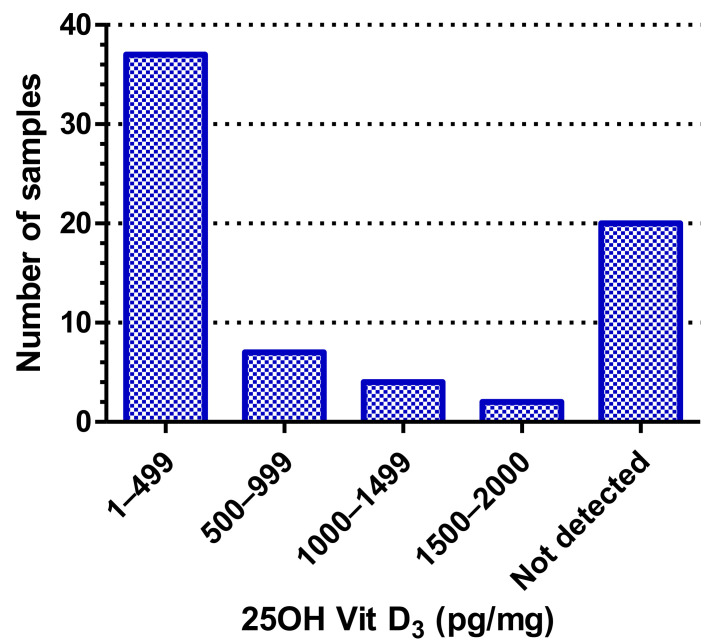
Prevalence and distribution of 25 hydroxyvitamin D_3_ levels in the hair of young Emirati males.

**Figure 3:**
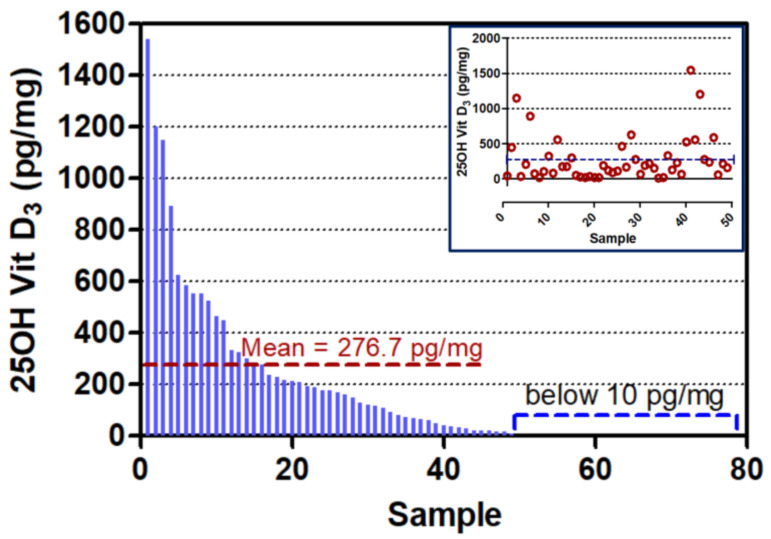
Individual 25 hydroxyvitamin D_3_ levels in the hair of 70 young Emirati males (where only 50 hair samples had detectable (>10 pg/mg) levels of 25OHD_3_). The inset shows the individual levels of 25OHD_3_ in the 50 samples.

This validated method was then applied on 70 hair samples (as described under Section 2) and the results are shown in Supplementary Table S1, Figure 2 and Figure 3. As can be seen from Table S1, of the eight forms of vitamin D screened for, we were only able to detect 25OHD_3_ in the hair samples. Additionally, as shown in Figure 2, there were around 20 samples that did not even show detectable levels of 25OHD_3_, indicating that they had less than 10 pg/mg levels of 25OHD_3_ in them. Of the remaining 50 samples, the majority (74%) had 25OHD_3_ ranging from 15–400 pg/mg, about 14% had between 25OHD_3_ between 500–999 pg/mg, around 8% of the samples had 25OHD_3_ between 1000–1499 pg/mg, and only two samples (4%) had high levels of 25OHD3 between 1500–2000 pg/mg (Figure 2). This distribution is shown in Figure 3, which also indicates the mean of the “Vit D_3_ positive” samples to be 276.7 pg/mg. The inset in Figure 3 shows the scatter of the 50 Vit. D_3_ positive samples.

Given that the assay can be used to quantify up to eight forms of vitamin D, it is feasible that, with a larger hair sample and pre-concentration steps, along with advances in instrument sensitivity, more forms of vitamin D could be quantified.

Recent increases in requests for vitamin D measurement are deemed to be costly and sometimes confusing [14]. The hair test, focusing on the major form 25OHD_3_, in corroboration with established parity with blood results, would allow clinicians to capture vitamin D levels in patients over the longer term at reasonable cost to use in conjunction with routine clinical tests. This ability to make a single measure of a quarterly average vitamin D level would overcome a number of the issues relating to vitamin D research. Further advantages include the non-invasive nature and lack of infection risk, along with easy storage and transportation of samples.

## 4. Further Steps

This pilot study revealed the potential for the development of a hair-based assay for vitamin D, which conveniently offers a quarter-year average for the major circulating form, which is currently the basis of most clinical assessments. Further work is warranted to develop and validate the procedure to include more metabolites of vitamin D and to produce a gold-standard test for hair analyses. With increasing interest in other vitamin D metabolites beyond the circulating forms, further developments should secure the ability to quantify as many forms as possible via concentration steps, using larger hair samples and more sensitive instrumentation, which is commercially available. Longitudinal studies correlating blood levels with hair levels would be required once the full range of analytes that can be measured in hair is established. This point is notable as there is widening interest in other metabolites in blood-based assays. A further point involves a further determination of the effect of age, hair color and type, and treatment on the analytes of interest. Observation of genetic variations that affect assay results such as those owing to the three major forms of vitamin D binding protein merit investigation [27,28]. It is envisaged that this approach will deliver a suitable test that will allow a single measure for the determination of sufficient/insufficient status in hair (in pg/mg) over many months. We also know that in UAE, people are generally deficient in vitamin D levels; that might be one reason that we were not able to find the remaining metabolites of vitamin D in hair. But this non-invasive hair test will be very useful in the segmental analysis of hair to measure vitamin D status over many months.

Limitations of this pilot study, apart from the small sample size, are the lack of a full spectrum of ethnicity-based hair types, different population groups, age groups, genders, seasonal variations, and color combinations. Hair from other parts of the human body and animals could also be investigated in future studies. Further research directions involve key questions relating to vitamin D levels in reproduction and contributions of inadequate levels to disease onset and progression. The ability of a vitamin D hair assay to monitor hypovitaminosis D and the ability of supplementation to normalize this condition would be clinically useful.

## Figures and Tables

**Table 1:** Retention times, MRM transitions, and collision energy conditions of each analyte.

Analytes	Retention Time (Min)	Precursor (*m*/*z*)	Product (*m*/*z*)	Collision Energy (eV)
Vitamin-D_3_	15.104	385	367	−13
		385	259	−16
		385	91	−55
Vitamin-D_2_	15.072	397.1	379.4	−17
		397.1	69	−29
25OHD_3_	6.98	383.2	365.3	−15
		383.2	107.1	−30
25OHD_2_	7.76	395.1	377.3	−17
		395.1	81.1	−38
3-epi-25OHD_3_	7.69	383.2	365.3	−15
		383.2	107.1	−30
3-epi-25OHD_2_	8.52	395.1	377.3	−17
		395.1	81.1	−38
1α25(OH)_2_ D_3_	3.82	399.1	381.3	−14
1α25(OH)_2_-D_2_	3.948	411.1	135.3	−13
		411.1	133.1	−12
IS [vitamin D_3_ (6,19,19-d3)]	6.97	386.35	368.25	−15
		386.35	257.2	−183

**Table 2:** Method validation results for intraday/interday precision, accuracy, and recovery.

Analytes	Conc. QC’s (pg/mg)	% Recovery	Intraday (*n* = 6)		Interday (*n* = 6)	
			Precision, % CV	Accuracy, %	Precision, % CV	Accuracy, %
Vitamin D_3_	200	79	3.23	100.1	4.82	99.8
	400	77	1.96	100.4	1.95	99.4
	1200	88	1.41	99.9	1.39	100.2
Vitamin D_2_	200	88	6.3	102.1	9.4	101.2
	400	89	10.2	98.5	7.1	99.8
	1200	102	8.6	99.4	3.2	101.3
25OHD_3_	200	86	4.9	99.9	3.4	101.1
	400	89	3.3	100.3	2.7	99.8
	1200	111	2.5	99.7	2.3	98.7
25OHD_2_	200	82	3.5	98.9	3.2	99.8
	400	99	4.3	99.4	3.3	98.9
	1200	104	2.9	99.9	2.3	97.8
1α25(OH)_2_ D_3_	200	75	3.5	107.2	4.7	100.5
	400	96	2.1	102.3	10.9	102.6
	1200	78	3.3	100.1	8.9	99.9
1α25(OH)_2_ D_2_	200	76	7.1	98.8	3.2	100.5
	400	87	5.9	99.6	2.7	100.1
	1200	89	3.2	89.9	3.2	100.3
3-epi-25OHD_3_	200	78	5.2	98.3	5.3	100.2
	400	89	2.3	102.6	2.1	103.3
	1200	97	4.1	100.9	3.6	108.1
3-epi-25OHD_2_	200	92	3.8	99.3	4.3	101.2
	400	94	3.5	98.7	2.1	99.8
	1200	97	3.2	101.2	3.2	99.9

## Data Availability

All relevant data are included in the paper or its Supplementary Information.

## Supplementary Materials

The following is available online: Table S1: Concentrations of various forms of vitamin D in the 70 hair samples.

## Sample Availability

Samples of the compounds are not available from the authors.
