# Supplementary material for: A Non-Invasive Hair Test to Determine Vitamin D3 Levels
Source: Molecules. 2021 May 28;26(11):3269. doi: 10.3390/molecules26113269 (PMC8198702; doi:10.3390/molecules26113269)
Supplement: Supplementary file 1 [file molecules-26-03269-s001.zip › molecules-1212192-supplementary.pdf]

Sample #	Vitamin D3	Vitamin D2	25OHD3	25OHD2	1α25(OH)2D3	1α25(OH)2D2	3-epi-25OHD3	3-epi-25OHD2
1	ND	ND	39	ND	ND	ND	ND	ND
2	ND	ND	ND	ND	ND	ND	ND	ND
3	ND	ND	449	ND	ND	ND	ND	ND
4	ND	ND	1150	ND	ND	ND	ND	ND
5	ND	ND	38	ND	ND	ND	ND	ND
6	ND	ND	209	ND	ND	ND	ND	ND
7	ND	ND	892	ND	ND	ND	ND	ND
8	ND	ND	ND	ND	ND	ND	ND	ND
9	ND	ND	ND	ND	ND	ND	ND	ND
10	ND	ND	74	ND	ND	ND	ND	ND
11	ND	ND	17	ND	ND	ND	ND	ND
12	ND	ND	107	ND	ND	ND	ND	ND
13	ND	ND	ND	ND	ND	ND	ND	ND
14	ND	ND	325	ND	ND	ND	ND	ND
15	ND	ND	81	ND	ND	ND	ND	ND
16	ND	ND	552	ND	ND	ND	ND	ND
17	ND	ND	175	ND	ND	ND	ND	ND
18	ND	ND	177	ND	ND	ND	ND	ND
19	ND	ND	ND	ND	ND	ND	ND	ND
20	ND	ND	ND	ND	ND	ND	ND	ND
21	ND	ND	299	ND	ND	ND	ND	ND
22	ND	ND	50	ND	ND	ND	ND	ND
23	ND	ND	30	ND	ND	ND	ND	ND
24	ND	ND	20	ND	ND	ND	ND	ND
25	ND	ND	33	ND	ND	ND	ND	ND
26	ND	ND	ND	ND	ND	ND	ND	ND
27	ND	ND	19	ND	ND	ND	ND	ND
28	ND	ND	20	ND	ND	ND	ND	ND
29	ND	ND	ND	ND	ND	ND	ND	ND
30	ND	ND	ND	ND	ND	ND	ND	ND
31	ND	ND	ND	ND	ND	ND	ND	ND
32	ND	ND	ND	ND	ND	ND	ND	ND
33	ND	ND	191	ND	ND	ND	ND	ND
34	ND	ND	122	ND	ND	ND	ND	ND
35	ND	ND	91	ND	ND	ND	ND	ND
36	ND	ND	115	ND	ND	ND	ND	ND
37	ND	ND	ND	ND	ND	ND	ND	ND
38	ND	ND	464	ND	ND	ND	ND	ND
39	ND	ND	ND	ND	ND	ND	ND	ND
40	ND	ND	168	ND	ND	ND	ND	ND
41	ND	ND	623	ND	ND	ND	ND	ND
42	ND	ND	275	ND	ND	ND	ND	ND
43	ND	ND	ND	ND	ND	ND	ND	ND
44	ND	ND	ND	ND	ND	ND	ND	ND
45	ND	ND	68	ND	ND	ND	ND	ND
46	ND	ND	188	ND	ND	ND	ND	ND
47	ND	ND	218	ND	ND	ND	ND	ND
48	ND	ND	147	ND	ND	ND	ND	ND
49	ND	ND	9.31	ND	ND	ND	ND	ND
50	ND	ND	17	ND	ND	ND	ND	ND
51	ND	ND	331	ND	ND	ND	ND	ND
52	ND	ND	130	ND	ND	ND	ND	ND
53	ND	ND	ND	ND	ND	ND	ND	ND
54	ND	ND	228	ND	ND	ND	ND	ND
55	ND	ND	66	ND	ND	ND	ND	ND
56	ND	ND	524	ND	ND	ND	ND	ND
57	ND	ND	1541	ND	ND	ND	ND	ND

58	ND	ND	552	ND	ND	ND	ND	ND
59	ND	ND	ND	ND	ND	ND	ND	ND
60	ND	ND	ND	ND	ND	ND	ND	ND
61	ND	ND	1201	ND	ND	ND	ND	ND
62	ND	ND	276	ND	ND	ND	ND	ND
63	ND	ND	ND	ND	ND	ND	ND	ND
64	ND	ND	235	ND	ND	ND	ND	ND
65	ND	ND	584	ND	ND	ND	ND	ND
66	ND	ND	60	ND	ND	ND	ND	ND
67	ND	ND	212	ND	ND	ND	ND	ND
68	ND	ND	161	ND	ND	ND	ND	ND
69	ND	ND	ND	ND	ND	ND	ND	ND
70	ND	ND	ND	ND	ND	ND	ND	ND

Table S1. Concentrations of various forms of vitamin D in the 70 hair samples. ND = below the detection limit.
